# The Role of Sartans in the Treatment of Stroke and Subarachnoid Hemorrhage: A Narrative Review of Preclinical and Clinical Studies

**DOI:** 10.3390/brainsci10030153

**Published:** 2020-03-07

**Authors:** Stefan Wanderer, Basil E. Grüter, Fabio Strange, Sivani Sivanrupan, Stefano Di Santo, Hans Rudolf Widmer, Javier Fandino, Serge Marbacher, Lukas Andereggen

**Affiliations:** 1 Department of Neurosurgery, Kantonsspital Aarau, 5001 Aarau, Switzerland; basil.grueter@ksa.ch (B.E.G.); fabio.strange@ksa.ch (F.S.); javier.fandino@ksa.ch (J.F.); serge.marbacher@ksa.ch (S.M.); lukas.andereggen@ksa.ch (L.A.); 2 Cerebrovascular Research Group, Neurosurgery, Department of BioMedical Research, University of Bern, 3008 Bern, Switzerland; sivani.sivanrupan@students.unibe.ch; 3 Department of Neurosurgery, Neurocenter and Regenerative Neuroscience Cluster, Inselspital, Bern University Hospital, University of Bern, 3010 Bern, Switzerland; stefano.disanto@insel.ch (S.D.S.); hansrudolf.widmer@insel.ch (H.R.W.)

**Keywords:** aneurysmal subarachnoid hemorrhage, delayed cerebral vasospasm, ischemic stroke, Sartans, therapeutic interventions

## Abstract

*Background*: Delayed cerebral vasospasm (DCVS) due to aneurysmal subarachnoid hemorrhage (aSAH) and its sequela, delayed cerebral ischemia (DCI), are associated with poor functional outcome. Endothelin-1 (ET-1) is known to play a major role in mediating cerebral vasoconstriction. Angiotensin-II-type-1-receptor antagonists such as Sartans may have a beneficial effect after aSAH by reducing DCVS due to crosstalk with the endothelin system. In this review, we discuss the role of Sartans in the treatment of stroke and their potential impact in aSAH. *Methods*: We conducted a literature research of the MEDLINE PubMed database in accordance with PRISMA criteria on articles published between 1980 to 2019 reviewing: “Sartans AND ischemic stroke”. Of 227 studies, 64 preclinical and 19 clinical trials fulfilled the eligibility criteria. *Results*: There was a positive effect of Sartans on ischemic stroke in both preclinical and clinical settings (attenuating ischemic brain damage, reducing cerebral inflammation and infarct size, increasing cerebral blood flow). In addition, Sartans reduced DCVS after aSAH in animal models by diminishing the effect of ET-1 mediated vasoconstriction (including cerebral inflammation and cerebral epileptogenic activity reduction, cerebral blood flow autoregulation restoration as well as pressure-dependent cerebral vasoconstriction). *Conclusion*: Thus, Sartans might play a key role in the treatment of patients with aSAH.

## 1. Introduction

Aneurysmal subarachnoid hemorrhage (aSAH) induces delayed cerebral vasospasm (DCVS) [1], cerebral inflammation [2,3], early brain injury [4], cortical spreading depression [5], delayed cerebral ischemia (DCI) [6], and lack of cerebral autoregulation [7] contributing to poor functional patients’ outcome. DCVS remains a major cause of patient’s morbidity and mortality by inducing delayed cerebral ischemia [8].

Multiple studies showed that endothelin-1 (ET-1), a most potent vasoconstrictor [9,10,11], plays a key role in the development of DCVS [12,13,14,15,16,17,18,19]. Although endothelin-A-receptor (ET_A_-R) antagonists in the treatment of DCVS in animal models are effective [10,20], clinical studies did not show beneficial effects [21,22]. It has been reported that the polypeptide angiotensin-II acts through two specific receptors, in essence the angiotensin-II-type-1- and angiotensin-II-type-2-receptor (AT_2_-1-R and AT_2_-2-R). Important to note is that activation of the AT_2_-1-R results in vasoconstriction while binding of angiotensin-II to the AT_2_-2-R causes vasorelaxation [23]. In line with this notion, preclinical as well as clinical trials showed promising results of Sartans, which are AT_2_-1-R antagonists, in ischemic stroke. Hence, Sartans may have a positive effect after aSAH by reducing DCVS due to crosstalk with the endothelin system. Thus, we aimed to analyze the potential role of Sartans in the treatment of aSAH.

## 2. Materials and Methods

We conducted a systematic literature research of the MEDLINE PubMed database in accordance with PRISMA guidelines on preclinical studies on the one and on clinical studies on the other hand published between 1980 to 2019 reviewing: “Sartans and ischemic stroke” [24]. Only articles in English were chosen for review. Search items with “Sartans” (*n* = 19,064) and “ischemic stroke” (*n* = 89,465) were extracted. For “Sartans AND ischemic stroke”, 227 publications met the inclusion criteria by excluding studies with commentary only, any duplicates, or results not commenting on cerebral effects of Sartans.

**Figure 1:**
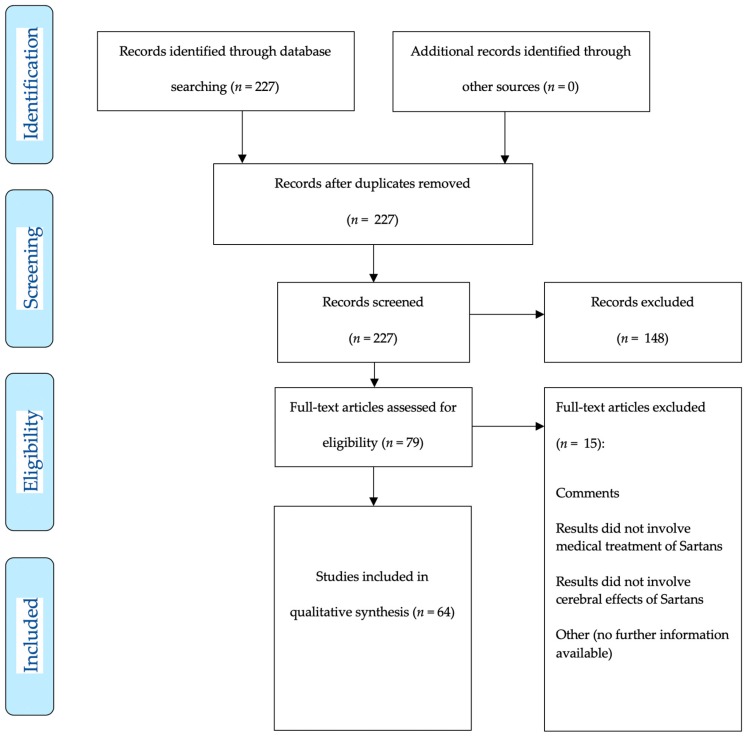
Two hundred and twenty-seven articles (published 01-01-1980–01-07-2019) were detected for preclinical and clinical research articles. After manual abstract screening for preclinical research articles only, 79 articles remained for further analysis. Each of the 79 articles was explicitly screened for potential drug applications after ischemic stroke. Finally, 64 articles were included for qualitative analysis.

**Figure 2:**
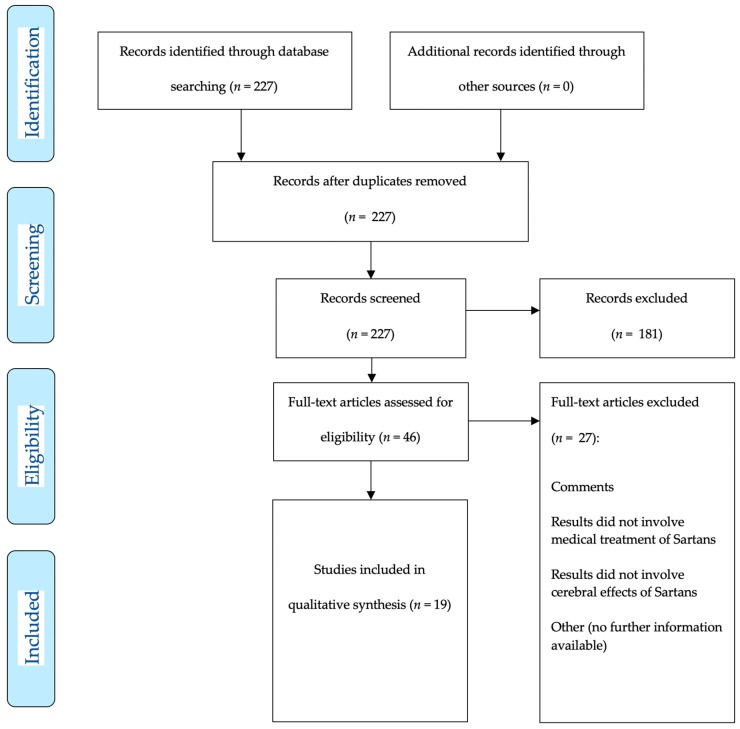
Two hundred and twenty-seven articles (published 01-01-1980–01-07-2019) were detected for preclinical and clinical research articles. After manual abstract screening for clinical research articles only, 46 articles remained for further analysis. Each of the 46 articles was explicitly screened for potential drug applications after ischemic stroke. Finally, 19 articles were included for final analysis.

Two hundred and twenty-seven studies were assessed for eligibility, 83 met inclusion criteria for systematic review and qualitative analysis with 64 preclinical studies (Figure 1 demonstrates the inclusion pathway for basic research studies selected via MEDLINE PubMed search) and 19 clinical studies (Figure 2 shows the inclusion pathway for clinical research studies selected via MEDLINE PubMed search).

Of the articles included in the final analysis, a systematic review on the beneficial and non-beneficial effect in the preclinical and clinical settings was performed. Summary measures are reported as outcome measures (i.e., infarct size, neurocognition, inflammation).

## 3. Results

### 3.1. Preclinical Studies on Sartans in Animal Models of Ischemic Stroke

The search finally yielded 64 preclinical studies on “Sartans AND ischemic stroke”, eligible for systematic review (Table 1).

Telmisartan (TMS), a selective AT_2_-1-R antagonist, displayed the capacity to increase cerebral blood flow (CBF) in global cerebral ischemia [25]. It ameliorated reduction of CBF in the penumbra (0.3 mg/kg/day) without significant changes in blood pressure (BP) [28]. Following middle cerebral artery occlusion (MCAO), TMS decreased ischemic infarct area, reduced superoxide production and expression of inflammatory cytokines, infiltration of inflammatory cells, improved neurological scores, and increased CBF [26,27]. Angiogenesis in ischemic areas after MCAO was enhanced by TMS, as well as neuroregeneration by downregulating caspase activation [29]. A combination of TMS with nimodipine (2.5–5 mg/kg) in a transient MCAO rat model revealed beneficial influences affecting the attenuation of excitatory amino acids in different brain regions nine days after MCAO with neurobehavioural outcomes normalized seven days after MCAO [31]. Low doses of TMS (0.3–3 mg/kg/d) after MCAO in a model of stroke-resistant spontaneously hypertensive rats (SR-SHR) reduced progressive decrease of N-acetylglucosamine oligomer and increase of MMP-9 positive neurons without reducing BP [32]. Likewise combination therapies with ramipril (0.8 mg/kg per day TMS + 0.1 mg/kg per day ramipril or 0.5 mg/kg per day TMS + 0.25 mg/kg per day ramipril) normalized BP as well as maintained cerebral blood flow autoregulation [33]. Deguchi et al. demonstrated that TMS dose-dependently (0.3 mg/kg/day or 3 mg/kg/day) ameliorated metabolic syndrome related changes in the post stroke brain of SR-SHR with direct neuroprotective effects [34]. Moreover, incidence of stroke was reduced along with prolonged survival and improved neurological outcome following TMS application (0.5 mg/kg once daily) [35]. Pretreatment of rats with TMS (1 mg/kg) seven days before inducing cerebral ischemia also showed significant reduced infarct size and histopathologically normal appearance of neurons in the periinfarct cortical regions [36].

Candesartan (CS), another AT_2_-1-R antagonist, reduced ischemic brain damage following MCAO occlusion [39]. CS and curcumin together significantly restored superoxide dismutase activity and blood flow compared with the untreated group [40]. Further, CS upregulated vascular endothelial growth factor (VEGF) B after induction of focal cerebral ischemia using a MCAO model. In contrast to saline-treatment after reperfusion, CS further improved neurobehavioral and motor functions and decreased infarct size [41]. VEGF-B silencing was shown to diminish CS (1 mg/kg) protective effects [42]. CS (0.3 mg/kg) was able to improve recovery from ischemic stroke in low doses by maintaining blood pressure during reperfusion [43]. CS induced early protective effects with improvement in motor function, upregulated brain-derived neutrotrophic factor (BDNF), and also reduced endoplasmatic reticulum stress markers [44]. In a MCAO BDNF, knock-out model rats received CS or saline at reperfusion for 14 days, revealing better functional outcomes, increased vascular density, and synaptogenesis in the CS (1 mg/kg) group [45,46]. In addition, CS (0.16 μM) significantly increased BDNF production [47]. Furthermore, CS (10 nM) improved cell function and viability of brain capillary endothelial cells under oxygen glucose deprivation, providing protective blood–brain-barrier (BBB) effects [48]. In other transient MCAO rat models, CS (0.1 mg/kg; 0.3 mg/kg; 1.5 or 10 mg/kg per day; 0.1, 1 and 10 mg/kg; 0.1, 0.3 or 1 mg/kg; 0.1 mg/kg twice daily; 1 mg/kg; 0.3 or 3 mg/kg per day; 0.5 mg/kg per day for 14 days; 0.1 or 0.3 mg/kg; 0.5 mg/kg per day for 3 to 14 days) showed improved neurological function with significant reduction in BBB disruption, in cerebral ischemia, and in edema [39,49,50,51,52,53,54,55,56,57,58,59,60]. In a bilateral common carotid artery occlusion (CCAO) model in rats, pretreatment with CS (0.1 and 0.3 mg/kg) and atorvastatin significantly attenuated neurobehavioral alterations, oxidative damage, and restored mitochondrial enzyme dysfunction compared to the control group [61,62]. AT_2_-1-R administration prior to ET-1 induced MCAO provides neuroprotective effects, with CS (0.2 mg/kg per day for seven days) pretreatment attenuating infarct size and neurological deficits without altering systemic BP [63]. Pretreatment with CS for five days significantly decreased mortality, neurological deficits, and infarct size [67]. A combined inhibition of AT_2_-1- (0.05 mg/kg per day) and ET_A_-receptors decreased brain damage as well; additionally, an upregulation of AT_2_-1-R in ischemic middle cerebral artery smooth muscle cells (SMCs) was found [68,69]. Also, early (3 h) and delayed (24 h) effects of CS treatment (0.3 and 3 mg/kg) continued for seven days after onset of MCAO with reperfusion in normotensive rats involved a reduction of the infarct volume by low doses of CS [70]. CBF in CS (0.5 mg/kg) pretreated animals at 0.5 h after MCAO was significantly increased compared to the control group [71]. Other groups additionally showed a four-week CS-pretreatment (0.3 mg/kg per day) before MCAO clearly associated with complete reversal of a decreased lumen diameter and increased media thickness as well as decreased endothelial nitric oxide synthase (eNOS) and increased inducible nitric oxide synthase (iNOS) protein and mRNA in SR-SHR and in a normotensive control group [72].

Olmesartan (OMS), an AT_2_-1-R antagonist, has been evaluated in a bilateral CCAO model in mice, revealing improved cognitive outcome, neuroprotective effects, attenuation of oxidative hippocampal stress, and suppression of BBB disruption compared to control groups [73]. A single carotid ligation stroke model in gerbils showed that OMS (10 mg/kg per day started 36 h after stroke) was associated with an increased survival [74]. Other studies demonstrated that OMS (10 mg/kg per day for 14 days after infarct; 10 mg/kg per day for 7 days before and 14 days after infarct; 10 mg/kg per day for 7 days before infarct) treatment in a rat MCAO model showed significantly better functional scores and reduced infarct size and cell death [75]. OMS (0.01 or 0.1 μmol/kg per hour for seven days) reduced brain angiotensin II, MMP-2 and MMP-9 upregulation following brain ischemia [77].

Valsartan (VS), a selective AT_2_-1-R antagonist, reduced ischemic brain area and improved the neurological deficit after MCAO with restoration of cerebral blood flow [78]. VS significantly reduced infarct volume and improved the neurological deficit scores. VS at nonhypotensive doses significantly diminished ischemic area, neurological deficits, and reduction of cerebral blood flow as well as superoxide production [27,78,80].

Irbesartan (IS), a selective AT_2_-1-R antagonist improved motor functions, reduced infarct size and decreased the number of apoptotic cells particularly in the periinfarct area by attenuated invasion of activated microglia likewise macrophages [82,83,84].

Losartan (LS), a clinical established selective AT_2_-1-R antagonist, did not increase mortality in acute cerebral ischemia [85]. Also, LS (20 μmol/L) abolished ischemic exaggeration of cell injury [26,86]. Expression levels of pro-apoptotic genes were significant reduced by LS treatment [87]. Further LS administration initiates cerebral angiogenic response with a significantly larger vessel surface area, and administration before initiation of cerebral focal ischemia (50 mg/day for 2 weeks) markedly reduces infarct size [88].

### 3.2. Clinical Studies on Sartans in Ischemic Stroke

The search yielded 19 clinical studies on “Sartans AND ischemic stroke”, eligible for systematic review (Table 2). Beneficial aspects of using AT_2_-1-R antagonists before the onset of ischemic stroke have already been elucidated in a retrospective analysis of 151 patients [89].

CS has been evaluated in the Scandinavian Candesartan Acute Stroke Trial (SCAST). Within 30 h of ischemic or hemorrhagic stroke, 2029 patients either received CS- or placebo-treatment. The modified ranking Scale (mRS) was used for outcome analysis. CS showed no overall effect on vascular events in ischemic and/or hemorrhagic stroke, and the adjusted odds ratio for vascular events of patients treated within 6 h reached significance [90]. At six months, activities of daily living and level of care were assessed. In more than 1800 patients, over 1500 suffered ischemic and almost 250 hemorrhagic strokes. No statistically significant effects of CS on Barthel index or level of care could be identified [91]. Furthermore, the SCAST group evaluated whether the effect of CS treatment varies in subtypes of over 1700 ischemic strokes. Concerning functional outcomes, a trend towards a beneficial effect of CS was observed in patients with larger infarcts (total anterior circulation or partial anterior circulation) than in patients with smaller lacunar infarcts [92]. Further on, over 2000 SCAST patients were randomly allocated to placebo or CS treatment for seven days with increasing doses from 4 mg (starting day 1) to 16 mg (from day 3 to 7). After six months’ follow-up, the risk of the composite vascular endpoint did not differ between the placebo and CS treatment group [93]. Also, the Acute Candesartan Cilexetil Therapy in Stroke Survivors study confirmed that administration of CS in the acute phase of stroke in 339 patients confers long-term benefits in patients who sustained acute ischemic stroke [94]. VS has been evaluated in a multicenter trial concerning efficacy and safety of modest blood pressure reduction within 48 h in more than 370 patients with acute ischemic stroke, considering the primary outcome death or dependency. The VS-treated group showed 46 of 187 patients with a 90-day mRS of 3–6, compared with 42 of 185 patients in the control group. The rate of major vascular events did not differ significantly between both groups [98]. TMS has also been evaluated concerning beneficial effects after stroke treatment. A multicenter trial, involving more than 18,500 patients with ischemic stroke, had a follow-up of 2.5 years. The primary outcome parameter was time to first recurrent stroke. Only short-term add-on TMS (80 mg/day) treatment did not mitigate this risk [100,101]. Treatment with TMS (80 mg/day) did not prevent progression of white matter lesions in patients with recent ischemic stroke [102]. Another study group enrolled 20,332 patients and analyzed 1360 patients within 72 h of ischemic stroke onset (TMS vs. placebo) concerning functional outcome after 30 days as primary outcome. Combined death or dependency did not differ between the treatment groups, showing treatment with TMS (80 mg/day) in patients with acute mild ischemic stroke and mildly elevated BP safe with no excess in adverse events [103]. Also, effects of TMS (80 mg/day) initiation early after stroke have been analyzed. From 20,332 patients with recent ischemic stroke, 10,146 patients were randomly assigned in the TMS group and 10,186 in the placebo group; 8.7% in the TMS group and 9.2% in the placebo group suffered from subsequent stroke, showing no significant reduction of recurrent stroke after early initiation [104]. LS has also been analyzed in recent clinical stroke trials. In a double-blinded multi-center trial, 196 hypertensive patients with previous ischemic stroke were randomized to cilnidipine- or LS-treatment (50–100 mg per day for four weeks) once daily for four weeks. Both treatments, however, increased global CBF despite BP lowering [105]. Additionally, the effect of long-term therapy with LS regarding cognitive function in 6206 essential hypertonic patients with additional cerebrovascular risk factors was investigated. The LS-based antihypertensive treatment increased the proportion of patients with normal cognitive function [109]. Also, the Losartan Intervention for Endpoint reduction in hypertension study group reported cardioprotective effects of a LS-based antihypertensive regimen. The incidence of any stroke, fatal stroke, and atherothrombotic stroke was significantly lower in LS-treated compared to the atenolol-treated isolated systolic hypertensive patients [106]. Other groups assessed the effect of LS treatment on mean arterial blood pressure, global, and focal CBF in 24 hypertensive patients without occlusive carotid disease 2–7 days after ischemic stroke and/or transient ischemic attack. LS (25–50 mg per day) was generally well tolerated and none of the patients suffered neurological deterioration. No changes occurred in internal carotid artery flow or cortical as well as hemispheric CBF [107].

### 3.3. Therapeutic Interventions After aSAH

Poor patients’ outcome after aSAH is owed a multifactorial process (early brain injury, DCVS, DCI, cerebral inflammation, cortical spreading depression, loss of pressure dependent cerebral autoregulation) [4,5,7,9,110,111,112,113]. DCVS is treated with moderate hypertensive, normovolemic, hemodilution, and in cases of therapy-refractory, DCVS with intra-arterial spasmolysis or balloon dilatation [114,115]. Research to improve poor functional outcome in patients suffering from aSAH and related DCVS is pivotal [1,5,21,116,117]. Multiple preclinical and clinical trials showed the effect of ET-1 in mediating DCVS after aSAH. CONSCIOUS-1, a randomized, double-blind, placebo-controlled study assessed the efficacy of intravenous clazosentan (ET_A_-R antagonist) in preventing vasospasm following aSAH. It significantly decreased angiographic DCVS with a trend for reduction in vasospasm-related morbidity/mortality [118]. CONSCIOUS-2 assigned patients with aSAH and clip ligation to clazosentan- or placebo. Thereby, clazosentan showed no significant difference in the mortality and vasospasm-related morbidity [119]. CONSCIOUS-3 assessed whether clazosentan reduced DCVS-related morbidity and mortality after aSAH and endovascular coiling. Pulmonary complications and anemia were more common in patients with clazosentan administration than in the placebo group, and mortality rates after 12 weeks were the same, respectively [120]. The REVERSE-study, infusing clazosentan intravenously in patients developing moderate to severe angiographic vasospasm after aSAH, showed a clear pharmacodynamic dilating effect on DCVS 24 h in most patients suffering aSAH, being able to reverse established angiographic vasospasm [22].

Antihypertensive agents are usually discontinued to maintain a sufficient mean arterial cerebral perfusion pressure considering the prolonged phase of DCVS between days 5 to 14 after the ictus [114]. In contrast, nimodipine, a calcium-channel antagonist, is administered for risk reduction of DCVS, yet rather its neuroprotective effects have been discussed in its beneficial role in aSAH [8,121].

### 3.4. Effects of Losartan Following aSAH

LS, an already well-established antihypertensive drug in daily clinical practice and well examined in preclinical and clinical settings of ischemic stroke, shows promising results by attenuating cerebral inflammation and restoring cerebral autoregulation [64,105,122,123,124,125]. Facing preclinical aSAH research, beneficial effects of Sartans have been shown. Under already physiological conditions, LS diminished cerebral inflammation and associated DCVS [126] as well as ET-1 mediated vasoconstriction. Targeted ET_B1_- and ET_A_-R-antagonism under LS administration revealed a direct modulatory ET_B1_-R dependent effect via inducing upregulation of the NO-pathway with a significantly increased relaxation accompanied with enhanced sensitivity of the ET_B1_-R [23]. After induction of aSAH, ET-1-induced vasoconstriction was likewise decreased by LS preincubation, abolished after pretreatment with an ET_B1_-R antagonist. In precontracted vessels with LS and ET_A_-R-antagonism, ET-1 induced a higher vasorelaxation compared to the control group without, clearly demonstrating a modulatory and functional restoring effect of LS on the normally after aSAH impaired ET_B1_-R function [127].

Beneficial effects of LS on ET-1- and PGF2α-mediated DCVS after aSAH in a rat model have been reported, too [23,127]. An ET-1 mediated vasoconstriction was diminished, and ET_B1_-R mediated vasorelaxation under selective ET_A_-R blockade was restored [126,127]. In addition, PGF2α-elicited vasoconstriction of a basilar artery was markedly diminished [23,126,127]. Interestingly, several work groups could also verify positive vasomodulating effects of LS on the cerebral vessel wall, especially affecting SMCs [128,129]. Furthermore, aneurysm rupture was prevented in mice under LS treatment [129]. As already mentioned, after aSAH, increased synthesis of ET-1 triggers enhanced cerebral vasoconstriction; loss of the ET_B1_-R mediated vasorelaxation contributes to this effect, too [127]. Furthermore, upregulated AT_2_-1-R and PGF2α-synthesis contribute in enhancing and maintaining cerebral vasocontraction [7,130,131,132,133]. LS showed promising aspects in preclinical aSAH studies and therefore might have an effect in the treatment of patients with aSAH.

## 4. Discussions

This systematic review demonstrated Sartan administration after ischemic stroke clearly associated with beneficial effects on preclinical models as well regarding clinical trials. Clear evidence of which doses in preclinical and clinical settings for treatment of ischemic stroke with Sartans exactly might be useful are heterogenous and therefore not consistent yet. In a preclinical setting, Sartans significantly reduced infarct volume and edema, augmented CBF, diminished superoxide production, inflammatory processes, and disruption of the BBB. In clinical studies, clear trends towards a better functional outcome and neurocognitive function after stroke with Sartan use have been reported. Thus, the question arises whether Sartans might provide positive effects on DCVS or DCI after aSAH. In summary, LS provided in a preclinical physiological and pathophysiological setup after aSAH beneficial aspects in reducing ET-1- and PGF2α mediated cerebral vasoconstriction [126,130]. Vasoconstriction was notably reduced and the vasorelaxant properties of the ET_B1_-R were restored. Furthermore, clear evidence exists, that after aSAH, AT_2_-1-R are upregulated in experimental settings [132]. Here, an additive direct antagonism on these receptors could reduce the sensitivity to an AT_2_-1-R-mediated vasocontraction to angiotensin II, too [125,134]. LS possesses beneficial aspects on cerebral epileptogenicity, which could be applied to the issue of reducing cortical spreading depression post aSAH [135,136,137,138]. Also, it is able to restore post-ischemic cerebral autoregulation after hemorrhagic stroke [134].

Considering these neuroprotective effects of LS, the ethical question arises of whether the philosophy of strictly discontinuing all antihypertensive agents after aSAH (except of new administration of nimodipine), especially of LS, should stay state of the art. Next to beneficial influences on DCVS after aSAH in rats as mentioned above, AT_2_-1-R antagonists clearly possess beneficial effects after stroke regarding cerebral inflammation, the areal of infarction, cortical spreading depression, cerebral microcirculation, and maintenance of pressure-dependent cerebral vasoconstriction [23,64,71,105,127,134,139,140,141]. Appreciating these facts, a systemic LS administration over and above the phase of DCVS, could be a promising approach in preventing these effects; particularly because LS seems to not influence the global CBF in essential hypertonic patients, which can be set equivalent to a needed-hypertonia after aSAH [142]. Here, LS could be an interesting approach, because it increases global CBF despite lowering blood pressure [105], and is therefore capable to reduce DCI [92]. Also, considering the positive vasomodulatory influences of LS, the question arises whether after aSAH this medication should be established as secondary prophylaxis to avoid a de-novo-aneurysm genesis, ergo, if aneurysms under LS are anyway arising [143].

### 4.1. Translational Aspects

Both abovementioned questions after aSAH are difficult to adapt to the affected patient group, because common sense to date stays in discontinuing all antihypertensive agents after the initial bleeding event. Also, it is vague to postulate that a LS effect persists after discharging this medication on admission over the phase of DCVS for 14 days. Furthermore, the numbers of patients with LS as standard antihypertonic medication receiving follow-up angiographys are too scarce to testify a valid statement concerning case-control studies of aneurysm-growth/-development, as reviewed in our own patient series in 2009–2015. Nevertheless, LS seems to be an underrated neuroprotective drug, reducing cerebral inflammation and epileptogenicity, DCVS, and infarct size after ischemic stroke. These results of preclinical ischemic stroke and aSAH research as well as clinical ischemic stroke research could be applied in a prospective clinical setting of patients suffering aSAH. Also, the question of a de-novo-aneurysm-genesis in further cranial control imaging could be addressed.

### 4.2. Synopsis and Forecast

LS, a selective AT_2_-1-R antagonist, was shown to directly antagonize and ameliorate the impaired ET_B1_-R vasodilatory function. Given that in most clinical centers, antihypertensive agents are discontinued during the period of DCVS, LS, although an antihypertensive drug, may have a role in preventing delayed DCVS after aneurysm rupture given the effects shown in ischemia. Following aSAH, immediate therapy with LS might antagonize the vasoconstrictive AT_2_-1-R without affecting the dilatory AT_2_-2-R effect [132,144,145,146,147,148,149,150,151]. Furthermore, AT_2_ interestingly increases endothelin production in non-cerebral vessels (an increased ET-1 concentration in rat aortas could be inhibited through LS administration [140]) and thus indirectly enhances ET-1-mediated DCVS [123,152,153,154,155,156]. All these aspects might suggest a crosstalk between both peptidergic systems extra- and intracranially [71,157].

## 5. Conclusions

There is a promising effect on LS in the treatment of ischemic stroke both in preclinical and clinical studies as well as in preclinical studies on aSAH. LS has shown to reduce ET-1-mediated vasocontraction, cerebral inflammation, and restores vasodilatory function of the ET_B1_-R [26,27,28]. Thus, LS may decrease the incidence of symptomatic vasospasm and improve functional outcome in aSAH patients. Large, randomized, double-blinded clinical trials are necessary to determine its benefit in aSAH.

## Figures and Tables

**Table 1:** Tabular listing of different preclinical studies showing various effects of Sartan administration (Abbreviations: Angiotensin-II-type-1-receptor (AT_2_-1-R); Angiotensin-II-type-2-receptor (AT_2_-2-R); common carotid artery occlusion (CCAO); chemokine receptor 2 (CCR2); cluster of differentiation (CD); candesartan (CS); desoxy ribonucleic acid (DNA); endothelial nitric oxide synthase (eNOS); endothelin-A-receptor (ET_A_-R); hours (h); inducible nitric oxide synthase (iNOS); irbesartan (IS); kilogram (kg); losartan (LS); middle cerebral artery occlusion (MCAO); matrix-metallo-proteinase (MMP); messenger ribonucleic acid (mRNA); milligram (mg); minutes (min); n-acetyl-glucosamine oligomer (NAGO); nlr family pyrin domain containing 3 (NLRP3); oxygen glucose deprivation (OGD); olmesartan (OMS); stroke-resistant spontaneously hypertensive rats (SR-SHR); telmisartan (TMS); tumor necrosis factor alpha (TNFα); ribonucleic acid (RNA); vascular endothelial growth factor (VEGF); valsartan (VS)).

Drug	Model	Outcome	Beneficial Effect	Special Remarks
TMS [25]	Global ischemic mice model	Cerebral perfusion	Restored cerebral blood flow	-
TMS [26]	MCAO mice	Neuroscore, infarct size	Improved neuroscore and decreased infarct size, increased cerebral blood flow, reduced superoxide production and inflammatory cytokine expression	-
TMS [27]	Murine model of transient and permanent focal ischemia	Infarct size, reperfusion injury	Reduced stroke volume 72 h after transient ischemia, likewise pro-inflammatory adhesion molecules and infiltration of inflammatory cells in the ischemic region	No reduction in stroke volume 72 h after permanent ischemia
TMS [28]	MCAO mice	Focal brain ischemia, atherosclerotic lesions	Attenuated ischemic brain damage, neurological deficits and superoxide production in ischemic area; attenuated reduction of cerebral blood flow in the penumbra without significantly changing blood pressure	Anti-atherosclerotic effects
TMS [29]	MCAO rat	Cerebral perfusion	Improved cerebral blood flow, enhanced vascular density (CD31 immunofluorescence staining), antiapoptotic effects	-
TMS [30]	MCAO rat	Cognitive function, level of matrix metalloproteinases	Improved spatial memory ability, decreased expression levels of MMP-2 and MMP-9	-
TMS [31]	MCAO rat	Behavior alterations, neuroprotective effects on secondary reperfusion phase	Normalized behavioral alterations comparable to pre-ischemic treatment (protected neurons from ischemic reperfusion injury), attenuated excitatory amino acid release in secondary reperfusion phase	In combination with nimodipine. Drug treatments immediately after reperfusion, effects compared with pretreatment
TMS [32]	MCAO rat	Effects on neurovascular unit and neuroinflammation	Reduced decrease of NAGO-positive endothelium, similar increase of MMP-9 positive neurons and NLRP3-positive inflammasome in the cerebral cortex	Low dose TMS improved changes without lowering blood pressure, high dose TMS further improved changes with lowering blood pressure
TMS [33]	Open skull preparation rat	Cerebral arteriolar pressure, cerebral blood flow, internal vessel diameter	Normalization of arteriolar pressure and lower limit of cerebral autoregulation	Combined with Ramipril
TMS [34]	MCAO rats	Metabolic related post-ischemic changes	Ameliorated metabolic related post-ischemic changes	-
TMS [35]	MCAO rats	Neurological outcome, infarct volume, inflammation	Improved outcome, reduced infarct volume and inflammation	Subcutaneous TMS application 5 days prior to MCAO with reperfusion
TMS [36]	MCAO rats	Infarct volume, immunohistochemical parameters	Significantly reduced infarct volume, reduced neurotoxic cytosolic phospholipase A2, ameliorates ischemic changes of neurons in the peri-infarct area	Pretreatment for 7 days
TMS [37]	Collagenase infusion or autologous blood injection to induce intracerebral hemorrhage in rats	Hemorrhage volume, functional recovery	Reduced hemorrhage volume, brain edema, inflammatory/apoptotic cells in perihematomal area; induced endothelial nitric-oxide-synthase, decreased oxidative stress, apoptotic signals, and TNFα	-
TMS [38]	Stroke-resistant spontaneously hypertensive rats	Oxidative stress	Reduced advanced glycation end product, 4-hydroxy-2-nonenal- and phosphorylated a-synuclein-positive cells in the cerebral cortex and hippocampus	-
CS [39]	MCAO mice	Ischemic brain damage	Reduced ischemic brain area and neurological deficits in non-hypotensive doses; improved reduction of brain surface blood flow and inhibited superoxide production in the cortex and brain arterial wall at non-hypotensive and hypotensive doses; AT_2_-2-R expression in the ischemic area was increased by prior pretreatment with CS	-
CS [40]	MCAO mice	Antioxidant enzyme activity	Restored superoxide dismutase activity and cerebral blood flow	-
CS [41]	MCAO rats	Neurobehavioral outcome, infarct size, vascular density	Improved neurobehavioral outcome, reduced infarct size and vascular density	In vitro vascular density was assessed using human brain endothelial cells
CS [42]	MCAO rats	Infarct size, neurological outcome	Improved neurobehavioral and motor functions, decreased infarct size	Intravenous CS administration
CS [43]	MCAO rats	Neurological outcome	Improved recovery from ischemic stroke	Only 0.3 mg/kg CS with neuroprotective function
CS [44]	MCAO rats	Neurological outcome, oxidative enzymes	Improved motor function and reduced endoplasmatic reticulum stress markers	Only early beneficial effect after 24 h
CS [45]	MCAO rats	Neurological outcome, vascular density/synaptogenesis	Improved functional outcome, increased vascular density/synaptogenesis only in the control group	Intracerebroventricular injection of short hairpin RNA lentiviral particles to knock down brain-derived neurotrophic factor or nontargeting control vector
CS [46]	MCAO rats	Angiogenesis	Induced prolonged proangiogenic effect and upregulation of VEGF-A and VEGF-B; stabilized hypoxia-inducible factor-1a and preserves angiopoetin-1	-
CS [47]	Spontaneously hypertensive rats	Angiogenesis	Exerted proangiogenic effects on brain microvascular endothelial cells	-
CS [48]	In vitro monolayer model using rat brain capillary endothelial cells	Stability of blood brain barrier	Improved cell function and viability of brain capillary endothelial cells under OGD	Normoxia versus 6 h OGD
CS [49]	MCAO rats	Neurological outcome, infarct size	Improved neurological function, significantly reduced blood brain barrier disruption/edema/infarct volume	-
CS [50]	MCAO rats	Infarct size, functional recovery, neuroplasticity	Significantly reduced infarct size, ameliorated functional recovery and increased neuroplasticity markers	-
CS [51]	MCAO rats	Infarct size, neurological outcome	Decreased infarct size and improved neurological outcome	-
CS [52]	MCAO rats	Mortality, infarct size	Significantly reduced mortality and infarct size	-
CS [53]	MCAO rats	Infarct size	Reduced infarct size	Oral administration
CS [54]	MCAO rats	Infarct size, edema, neurological outcome	Reduced infarct size, edema formation and improves neurological outcome	-
CS [55]	MCAO rats	Infarct size, neurological outcome	Significantly reduced stroke volume and improved neurological outcome	-
CS [56]	MCAO rats	Infarct size, edema	Reduced infarct size and edema, improved neurologic function	-
CS [57]	MCAO rats	Infarct volume, neurological deficit	Reduced infarct size and improved neurologic outcome	-
CS [58]	MCAO rats	Infarct volume, neurological deficits	Reduced infarct size, improved neurological outcome, reduced lipid peroxidation	Subcutaneous infusion for 14 days
CS [59]	MCAO rats	Infarct volume, neurological deficits	Reduced infarct size/edema and improved neurological outcome	Long-term blockade (subcutaneous injection twice daily 5 days before ischemia), not short-term administration (intravenous once 4 h prior to ischemia), improves neurological outcome
CS [60]	MCAO rats	Infarct volume, brain edema	Significantly reduced cortical infarct volume and brain edema	-
CS [61]	Bilateral CCAO rats	Neurological outcome, oxidative damage	Attenuated neurobehavioral alterations, oxidative damage and restored mitochondrial enzyme dysfunction	Occlusion for 30 min, followed by 24 h reperfusion; CS pretreatment for 7 days
CS [62]	MCAO rats	Infarct size	Reduced infarct area	-
CS [63]	MCAO rats	Infarct size, neurological outcome	Pretreatment reduced infarct area and improved neurological outcome	-
CS [64]	MCAO rats	Infarct size, neurological outcome	Reduced infarct size and neurological deficits; significantly reduced mRNA expression of inflammatory markers	-
CS [65]	Spontaneously hypertensive rats	AT_2_-1-R expression	Increased AT_2_-2-R expression in spontaneously hypertensive rats	CS application via subcutaneous osmotic minipumps for 4 weeks
CS [66]	MCAO rats	Neurological outcome, vascular density	Improved neurological outcome and increased vascular density	-
CS [67]	Embolic stroke model	Mortality, neurological outcome, infarct size	Significantly decreased mortality, neurological deficits, and infarct size	Injection of calibrated microspheres
CS [68]	MCAO rat	Infarct size, neurological outcome	Reduced infarct size and improved neurological outcome	Combined treatment with ET_A_-R antagonist
CS [69]	MCAO rats	Contractile response to angiontensin II	Abolished the enhanced responses to angiotensin II	-
CS [70]	MCAO rats	Infarct volume, neurological outcome	Reduced infarct size with low but not high dose of CS, improved neurological outcome	Subcutaneous CS administration
CS [71]	MCAO rats	Infarct size, neuroscores, cerebral blood flow	Reduced infarct size and increased cerebral blood flow	Intravenous CS administration
CS [72]	Spontaneously hypertensive rats	Vascular remodeling, expression of eNOS/iNOS	Reversed negative vascular remodeling and alterations in eNOS/iNOS expression	-
OMS [73]	Bilateral CCAO mice	Cognitive impairment	Ameliorated cognitive impairment	-
OMS [74]	Single carotid ligation stroke model gerbil	Survival	Significantly increased survival at day 30	-
OMS [75]	MCAO rats	Neurological outcome, infarct size, cell death	Significantly improved functional scores, reduced infarct size and cell death	Only continuous administration of OMS before and after stroke reduced oxidative stress levels
OMS [76]	MCAO rats	Infarct volume	Reduced infarct volume 48 h after transient focal brain ischemia	OMS administration via drinking water
OMS [77]	MCAO rats	Stroke index score, infarct volume, quantity of MMPs	Improved stroke index score, infarct volume, reduced cerebral edema and upregulation of MMPs	-
VS [78]	MCAO mice	Infarct volume, DNA damage, superoxide production	Significantly reduced infarct size, DNA damage, superoxide production, mRNA levels of monocyte chemoattractant protein-1, increases cerebral blood flow, increased eNOS activation and nitric oxide production	-
VS [79]	MCAO mice	Infarct volume, neurological outcome	Significantly reduced infarct volume and improved neurological outcome	-
VS [80]	MCAO mice	Infarct volume, neurological outcome	Significantly reduced ischemic area, neurological deficits, reduction of cerebral blood flow and superoxide production	-
VS [81]	High salt loaded SR-SHR	Brain injury	Enhanced protective effects against brain injury, white matter lesions and glial activation	Combined with amlodipine
IS [82]	MCAO rats	Infarct size, neurological outcome	Reduced infarct size and number of apoptotic cells in the peri-infarct cortex on day 3, attenuated invasion of microglia and macrophages on day 3 and 7 after ischemia	-
IS [83]	MCAO rats	Neurological outcome	Significantly improved neurological outcome	Administration of IS intracerebroventricularly over 5 days
IS [84]	MCAO rats	Infarct size	Reduced infarct volume	Coadministration of propagermanium (CCR2 antagonist)
LS [85]	Single carotid ligation stroke model gerbil	Mortality	Did not increase mortality after unilateral carotid ligation in gerbils	-
LS [86]	MCAO mice	OGD-induced cell injury	Abolished OGD-induced exaggeration of cell injury in mice overexpressing renin and angiotensinogen animals	-
LS [87]	MCAO rats	Gene expression levels of pro-apoptotic genes	Significant reduced gene expression of pro-apoptotic genes	-
LS [88]	Cerebral focal ischemia by cauterization of cortical surface vessels rats	Cessation of blood flow, infarct size	Maintained angiogenesis, vascular delivery, and significantly decreased infarct size	Administration of LS in drinking water 2 weeks before inducing ischemia

**Table 2:** Tabular listing of different clinical studies showing various effects of Sartan administration (Abbreviations: Candesartan (CS); hours (h); losartan (LS); milligram (mg); minutes (min); μmol (micromolar); modified ranking Scale (mRS); mol (molar); nmol (nanomolar); telmisartan (TMS); valsartan (VS)).

Drug	Outcome	Beneficial Effect	Special Remarks
CS [90]	Vascular event (vascular death, nonfatal stroke or nonfatal myocardial infarction) over 6 months and mRS	No overall effect on vascular events in ischemic and/or hemorrhagic stroke, adjusted odds ratio for vascular events of patients treated within 6 h reached significance	Administration at least within 30 h of ischemic or hemorrhagic stroke. CS treatment for 7 days, increasing from 4 mg on day 1 to 16 mg on day 3 to 7
CS [91]	Barthel index and level of care assessed after 6 months	No significant effects on Barthel Index or level of care at 6 months	Administration at least within 30 h of ischemic or hemorrhagic stroke. CS treatment for 7 days, increasing from 4 mg on day 1 to 16 mg on day 3 to 7
CS [92]	Vascular death, myocardial infarction, stroke during first 6 months and functional outcome at 6 months	Significant trend towards a better effect of CS in patients with larger infarcts; no differences in treatment effect for composite vascular end point	CS treatment for 7 days, increasing from 4 mg on day 1 to 16 mg on day 3 to 7
CS [93]	Vascular death, myocardial infarction, stroke during first 6 months and functional outcome at 6 months	After 6 months the risk of the composite vascular endpoint did not differ between treatment groups	CS treatment for 7 days, increasing from 4 mg on day 1 to 16 mg on day 3 to 7
CS [94]	Safety of modest blood pressure reduction by CS cilexetil in the early treatment of stroke	The cumulative 12 months mortality and the number of vascular events differed significantly in favor of the CS cilexetil group	CS treatment with 4 mg on day 1; dosage increased to 8 mg on day 2 or 16 mg if blood pressure exceeded 160 mmHg systolic or 100 mmHg diastolic
CS [95]	Short-term safety of blood pressure reduction in hypertensive patients with acute ischemic stroke	CS treatment safely reduces blood pressure in hypertensive patients with acute ischemic stroke	4 mg/day for 14 days
CS [96]	Adhesion of neutrophils to human endothelial cells in acute ischemic stroke	CS inhibited the adhesion of neutrophils to vascular endothelium in ischemic stroke patients (not in chronic stroke patients or healthy volunteers)	Incubation with 10^−9^ mol for 30 min
CS [97]	Effect of blood pressure lowering in patients with acute ischemic stroke and carotid artery stenosis (Vascular death, stroke, myocardial infarction, and functional outcome at 6 months)	No evidence that CS effect is qualitatively different in patients with carotid artery stenosis	CS treatment for 7 days, increasing from 4 mg on day 1 to 16 mg on day 3 to 7
VS [98]	Safety of modest blood pressure reduction within 48 h of acute ischemic stroke	After 90 days the mRS as well the rate of major vascular events differed not significantly between both groups	80 mg/day (dose was modified in the subsequent six-days of treatment if the target systolic blood pressure was not achieved)
VS [99]	Effect of vs. on human platelet aggregation	VS exhibited significant inhibition of human platelets and therefore might be able to reduce vascular ischemic events	10 nmol to 100 μmol
TMS [100]	Time to first recurrent stroke	Low glomerular filtration rate (<60 mL/min) is independently associated with a higher risk of recurrent stroke, TMS not able to mitigate this risk	TMS dosage not reported
TMS [101]	Recurrent stroke of any type	Similar rates of recurrent strokes comparing aspirin plus extended-release dipyridamole with clopidogrel and TMS	80 mg/day
TMS [102]	Prevention of cerebral white matter lesions	TMS on top of existing antihypertensive medication did not prevent the progression of white matter lesions	80 mg/day. Analysis limited by the relatively short follow-up
TMS [103]	Functional outcome at 30 days (primary outcome), death, recurrence, and hemodynamic measures up to 90 days (secondary outcomes)	TMS treatment appears to be safe with no excess in adverse events and not associated with a significant effect on functional dependency, death, or stroke recurrence	80 mg/day
TMS [104]	Recurrent stroke	TMS initiated soon after ischemic stroke and continued for 2.5 years did not significantly lower the rate of recurrent stroke, major cardiovascular events, or diabetes	80 mg/day
LS [105]	Global change of cerebral blood flow	LS treatment increases the global cerebral blood flow despite blood pressure lowering	50–100 mg/day for 4 weeks
LS [106]	Effect on stroke in patients with isolated systolic hypertension and left ventricular hypertrophy	Incidence of any stroke (40% risk reduction), fatal stroke (70% risk reduction), and atherothrombotic stroke (45% risk reduction) was significantly lower in the LS treated group compared to atenolol treated patients	Mean LS dose of 79 mg
LS [107]	Effect on global and focal cerebral blood flow in hypertensive patients 2–7 days after stroke	No neurological deterioration in the LS group	25–50 mg/day
LS [108]	Spontaneous platelet aggregation and P-selectin levels (in patients with hypertension and chronic ischemic stroke)	Spontaneous platelet aggregation was not, P-selectin levels significantly reduced after LS treatment. This suggests that standard doses of LS display antiplatelet effect	50 mg/day
